# Microglial Involvement in Neuroplastic Changes Following Focal Brain Ischemia in Rats

**DOI:** 10.1371/journal.pone.0008101

**Published:** 2009-12-01

**Authors:** Alexandre Madinier, Nathalie Bertrand, Claude Mossiat, Anne Prigent-Tessier, Alain Beley, Christine Marie, Philippe Garnier

**Affiliations:** 1 Unité INSERM U887 Motricité-Plasticité, Dijon, France; 2 Université de Bourgogne, Dijon, France; 3 Département Génie Biologique, IUT, Dijon, France; University of North Dakota, United States of America

## Abstract

The pathogenesis of ischemic stroke is a complex sequence of events including inflammatory reaction, for which the microglia appears to be a major cellular contributor. However, whether post-ischemic activation of microglial cells has beneficial or detrimental effects remains to be elucidated, in particular on long term brain plasticity events. The objective of our study was to determine, through modulation of post-stroke inflammatory response, to what extent microglial cells are involved in some specific events of neuronal plasticity, neurite outgrowth and synaptogenesis. Since microglia is a source of neurotrophic factors, the identification of the brain-derived neurophic factor (BDNF) as possible molecular actor involved in these events was also attempted. As a means of down-regulating the microglial response induced by ischemia, 3-aminobenzamide (3-AB, 90 mg/kg, i.p.) was used to inhibit the poly(ADP-ribose) polymerase-1 (PARP-1). Indeed, PARP-1 contributes to the activation of the transcription factor NF-kB, which is essential to the upregulation of proinflammatory genes, in particular responsible for microglial activation/proliferation. Experiments were conducted in rats subjected to photothrombotic ischemia which leads to a strong and early microglial cells activation/proliferation followed by an infiltration of macrophages within the cortical lesion, events evaluated at serial time points up to 1 month post-ictus by immunostaining for OX-42 and ED-1. Our most striking finding was that the decrease in acute microglial activation induced by 3-AB was associated with a long term down-regulation of two neuronal plasticity proteins expression, synaptophysin (marker of synaptogenesis) and GAP-43 (marker of neuritogenesis) as well as to a significant decrease in tissue BDNF production. Thus, our data argue in favour of a supportive role for microglia in brain neuroplasticity stimulation possibly through BDNF production, suggesting that a targeted protection of microglial cells could represent an innovative approach to potentiate post-stroke neuroregeneration.

## Introduction

The pathogenesis of ischemic stroke is a complex sequence of events including inflammatory reaction. Extensively studied over the last decade, this phenomenon is characterized by the involvement of several central and peripheral cell types as well as a large number of inflammatory molecules [1]–[3]. Post-ischemic inflammation includes the infiltration of polymorphonuclear granulocytes, monocytes/macrophages into the injured brain and the activation of astrocytes and microglia. Among these cells, it is now well admitted that microglia appears to be a major cellular contributor of post-ischemic inflammation [4]. After focal ischemia, reactive microgliosis is characterized by a specific chronology which includes a rapid microglial activation followed by a massive expansion and migration of the resident microglial cells. Several studies have shown that this initial intrinsic response is followed by the recruitment of blood-born macrophages which migrate after a delay of several days into the neuronal parenchyma [5]–[7].

Whether microglial activation has beneficial or detrimental effects on adjacent neuronal population is still controversially discussed [8]–[10]. It has been proposed that these cells, through the release of several harmful components such as IL-1β, TNF-α, proteases and ROS species [11]–[14] can affect neuronal function and promote neurotoxicity [1], [11], [15]. In addition, reducing inflammatory response and microglial activation has conferred neuroprotection in various models of neurodegeneration [16], [17] and has been shown to interfere with neurogenesis [18], [19]. On the other hand, there are also growing evidences showing that under certain circumstances, microglia could be neuroprotective [20]–[22] and could also promote adult neurogenesis [23], [24]. Indeed, microglia has been shown to be neurosupportive by the uptake of glutamate [25], the removal of cell debris [26] and recently by the ungulfment of polymorphonuclear neutrophiles [27]. In addition, beyond this scavenger function, several evidences have demonstrated that once activated or in proliferation, microglial cells are also an important cellular source for the production of neurotrophic factors such as IGF-1 [22], [24] and BDNF [28]. Concerning this latter, evidences showing microglia as a source of BDNF have been reported in *in vitro* studies [29] and after CNS injuries such as traumatic brain injury [30] and striatal lesion [28]. To the best of our knowledge, there are surprisingly no data identifying microglia expressing BDNF after *in vivo* models of focal ischemia although this trophic factor has been designated to play a central role in the CNS, as neuroprotective [31], [32] and essential to the stimulation of brain plasticity [33]–[35].

Thus, despite important progress in the understanding of microglial activation, proliferation, phagocytosis function, cytokines and growth factors production, the exact role of microglia is still unclear. Even though several studies have been performed in order to determine the function of these cells in neurogenesis [10], little is known concerning the role of microglia in other long term post-stroke brain plasticity events. In this context, the objective of our study was to determine through modulation of inflammatory response, to what extent microglial cells are involved in some specific events of neuronal plasticity such as neurite outgrowth and synaptogenesis. In addition, the identification of the neurotrophin BDNF as possible molecular actor involved in these events was attempted following ischemic injury. For this purpose, rats were subjected to a photothrombotic ischemic stroke which is associated with a strong and early microglial cells activation/proliferation within the cortical lesion. The modulation of the inflammatory response was performed by using a poly(ADP-ribose) polymerase-1 (PARP-1) inhibitor [3-aminobenzamide (3-AB), 90 mg/kg, i.p.]. Indeed, beside its function in DNA repair and its well established participation in cell death through extensive activation after stroke [36], PARP-1 has been shown to contribute to the activation of transcription factors, among them the most important NF-kB that has been designated to be essential to the upregulation of proinflammatory genes and to lead to microglial activation/proliferation [37], [38]. Activated microglia was evaluated at serial time points up to 1 month after ischemia by immunostaining for OX-42 and ED-1. In parallel, the long term expression of two neuronal plasticity proteins, GAP-43 as a marker of neuritogenesis and synaptophysin as a marker of synaptogenesis as well as the tissue production and cellular localization of BDNF were also analyzed.

## Materials and Methods

### Materials

3-Aminobenzamide (3-AB), protease inhibitor cocktail, phosphate buffered saline (PBS), tris buffered saline (TBS) and all other reagent grade chemicals were purchased from Sigma (Saint Quentin-Fallavier, France). Acrylamide and bis-acrylamide, sodium dodecylsulfate (SDS) and tween-20 were obtained from Bio-Rad (Ivry sur Seine, France). The polyvinylidene difluoride (PVDF) membranes were purchased from GE Healthcare (Orsay, France). The rabbit polyclonal antibody raised against synaptophysin (RB-1461-P) and the mouse monoclonal antibody recognizing rat GAP-43 (Zymed 33–5000) were purchased respectively from Interchim (Montluçon, France) and Invitrogen (Cergy-Pontoise, France). The mouse monoclonal CD11b (OX-42) that recognizes type 3 complement receptors (MCA 275R) and the mouse monoclonal ED-1 raised against rat lysosomal membrane antigen of activated macrophage/microglia were obtained from AbD Serotec (Darmstadt, Germany). Rabbit polyclonal anti-PAR recognizing poly(ADP)-ribose polymers and anti-BNDF were purchased from Calbiochem (Meudon, France) and Chemicon (Molsheim, France), respectively. For secondary antibodies, the Alexa Fluor 488- and 568-conjugated anti-rabbit and –mouse antibodies were purchased from Molecular probes and the horseradish peroxidase-conjugated anti-rabbit and –mouse antibodies were obtained from Jackson ImmunoResearch Laboratories.

### Animal Model

The experiments were carried out on 132 male Wistar rats (290–310 g; Depré, Saint-Doulchard, France) and were conducted according to the French Department of Agriculture guidelines (license no. 21CAE035). The animals were housed five per cage and kept under 12/12 h light/dark cycle and allowed *ad libitum* access to food and water. Anesthesia was induced by i.p. injection of chloral hydrate (400 mg/kg). Permanent focal ischemia was induced by photothrombotic cortical occlusion as previously described [39]. Briefly, anesthetized rats were infused for 20 s with the photosensitizer dye rose bengal (20 mg/kg, i.v.) and a laser beam was focused with an optic fiber (1 mm internal diameter, emerging power 90 mW) through the skull on the right hemisphere (1 mm posterior and 3 mm lateral relative to the bregma). The laser system is a diode-pumped solid-state laser (LCS-DLT-312, Opton Laser International, Orsay, France) working at 532 nm. The skull was irradiated for 5 min, the irradiation beginning 30 s before the dye injection.

### Cortical Tissue Sampling

**Figure 1:**
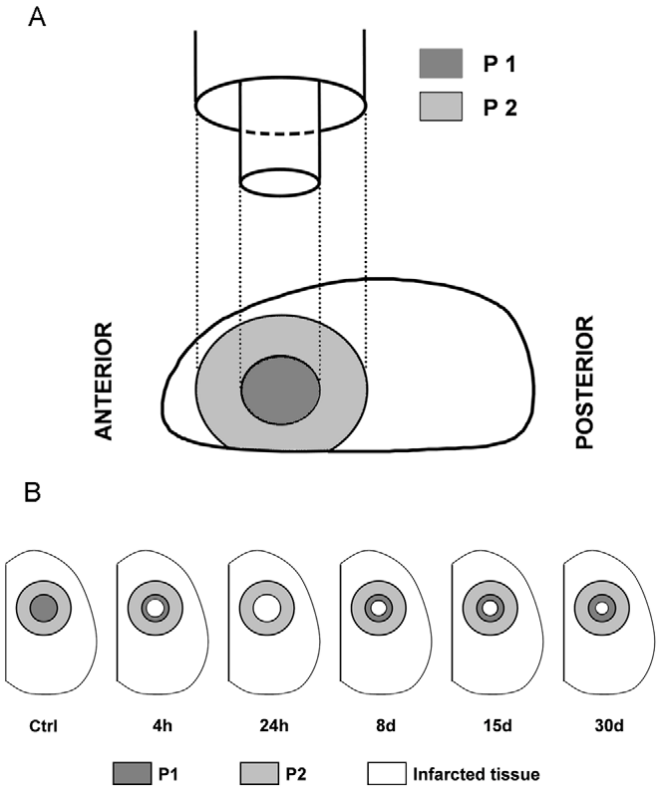
Cortical tissue sampling and dimensions of ischemic lesion. (A) Two concentric cortical samples (P1 and P2) were collected using punches with increasing internal diameters (4,6 and 9,5 mm, respectively). (B) At 24 h, P1 corresponded to lesioned tissue whereas P2 rim corresponded to unlesioned tissue whatever the time point considered. Infarcted area (Blank zone) evolves over the time after focal photothrombotic ischemia. Ctrl = control.

Anesthetized animals (400 mg/kg; chloral hydrate; i.p.) were transcardially perfused with saline and then, brains were removed. The cerebral cortices were quickly dissected and spread on glass slide at 0°C. Two concentric samples of cortical tissue (P1, P2) were punched with increasing internal diameter (4.6 and 9.5 mm) as indicated in figure 1A. The diameter of the different punches was chosen from the histological assessment of the ischemic lesion [40]. At 24 h post-ictus when the lesion reaches maximal size, P1 corresponded to infarcted tissue only. At other post-ictus times, P1 corresponded to a mixed of infarcted and non infarcted tissue while the surrounding rim P2 corresponded to unlesioned tissue whatever the time point considered as shown in figure 1B.

### Immunoblot Analysis

After homogenization in seven volumes of lysis buffer [bi-distilled water containing 50 mM Tris, 150 mM NaCl, 1 mM EGTA, 10% glycerol, 1% triton X-100, 1% protease inhibitor (Sigma P8340)], total protein extracts of the different punches were sonicated and centrifuged at 10,000×*g* for 10 min. An aliquot of the supernatant was kept for BCA protein measurement. Equal amounts of proteins were dissolved in Laemmli solution (62.5 mM Tris–HCl (pH 6.8), 2% SDS, 10% glycerol, 0.001% bromophenol blue) with 2-mercaptoethanol 5% and were heated at 85°C for 10 min. Proteins were separated on 10–12% SDS–polyacrylamide gel electrophoresis (PAGE) according to [41]. Proteins were electrophoretically transferred onto PVDF membrane (0.2 µm pore size) in cold transfer buffer [10 mM NaHCO_3_, 3 mM Na_2_CO_3_ (pH 9.9) and 20% methanol]. The membranes were incubated overnight at 4°C in 5% non-fat dry milk in TBS [20 mM Tris–HCl (pH 7.6) and 137 mM NaCl] containing 0.1% Tween 20 to block unspecific binding. Membranes were washed, incubated for 4 h at room temperature with specific primary antibodies at the following dilutions: anti-synaptophysin (1/2000), anti-GAP-43 (1/2000) and anti-β-actin (1/5000) and for 90 min with a horseradish peroxidase (HRP)-conjugated anti-mouse IgG or anti-rabbit IgG (1/80000). Protein–antibody complexes were visualized using the enhanced chemiluminescence Western blotting detection system according to the manufacturer's protocol (GE Healthcare, Orsay, France). The membranes were stripped and probed with an anti-β-actin antibody (Sigma A5441) used as internal control. Synaptophysin, GAP-43 and β-actin band densities were determined by scanning densitometry (Vilbert-Lourmat, Marne la Vallée, France). A computer-based imaging system (Scion, NIH software, Bethesda, USA) was used to measure the relative optical density of each specific band. Data were expressed as arbitrary units.

### Elisa

Cerebral levels of BDNF were determined with a commercial ELISA kit (Chemikine, Chemicon, Molsheim, France) according to the manufacturer's instructions. Briefly, after dilution in the homogenization buffer (1/10, v/v, see above for its composition), 50 µl of cerebral samples obtained from P1 and P2 punches were incubated overnight in pre-coated microplates. They were then incubated with biotinylated anti-BDNF antibody followed by HRP-streptavidin. The oxidation of the enzyme substrate, the 3,3′,5,5′ tetramethylbenzidine (TMB), was measured at 450 nm using a plate reader (vector-3 1420 multilabe, Perkin Elmer, Waltham, USA). All assays were performed in triplicate. Cortical BDNF levels were expressed in pg/mg of proteins.

### Histology

Animals were anaesthetized with chloral hydrate (400 mg/kg, i.p.) and the brains were washed by transcardiac perfusion with saline and further perfused with 4% paraformaldehyde solution in 0.1 M phosphate buffer (pH 7.5). The removed brains were postfixed in the same fixative for 1 h and transferred for 48 h in a 20% sucrose solution in PBS. They were then frozen in isopentane at −45°C and stored at −80°C. Coronal sections (20-µm thick) were cut in a cryostat (HMSSO, Microm, Francheville, France) at −20°C.

### Assessment of the Infarct Volume

**Figure 2:**
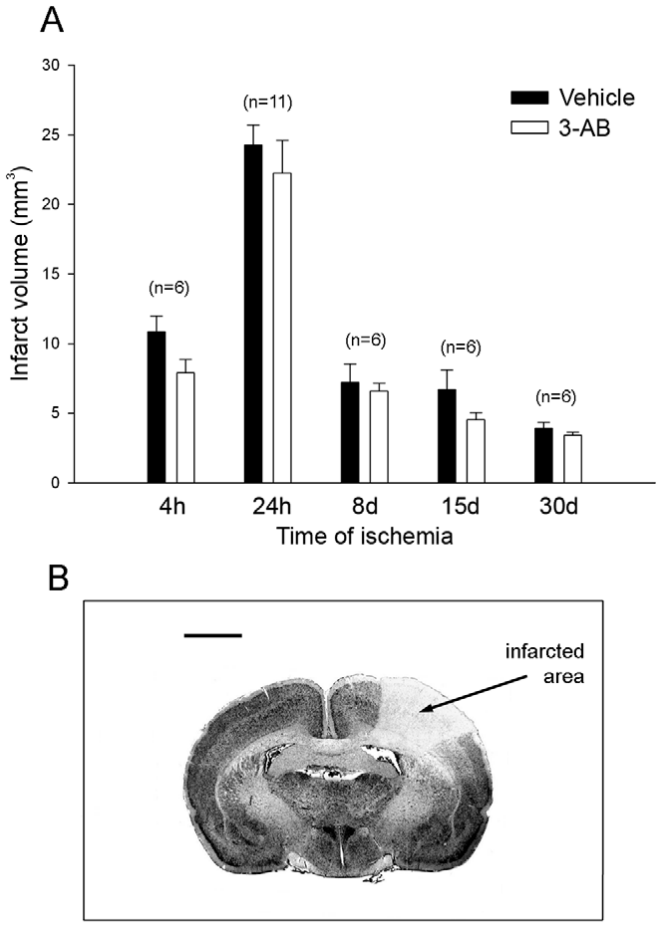
Temporal evolution of infarct volume: effect of 3-AB treatment. (A) Measurements of infarct volume (mm^3^) were performed at 4 h, 24 h, 8 d, 15 d and 30 d after the onset of ischemia. 3-AB (90 mg/kg) was given (i.p.) right after the induction of photothrombotic ischemia. Values are expressed as means±S.E.M. n in bracket indicates the number of animals in each experimental condition. (B) Representative photograph of a brain section stained with Cresyl violet after 24 h of ischemia. The injured cortical area is unstained as the reflection of cell loss. Scale bar = 2.5 mm.

Coronal sections (20-µm thick) were cut in a cryostat at 200 µm intervals, collected on slides, and stained with Cresyl violet (0.4%, pH 3.5). Injured cortical areas, i.e. unstained tissue as a reflection of cell loss (Figure 2B), were measured using the computer image analysis system (Scion, NIH software, Bethesda, USA) and the distance between respective coronal sections were used to calculate a linear integration for the lesion volume determination as previously described [42].

### Immunohistofluorescence

All brain sections from different animal groups were simultaneously run to ensure identical staining conditions. After rinsing in PBS (pH 7.4), the sections were incubated for 2 h in 10% goat serum (GS), 0.3% Tween-20 in PBS to block the non-specific binding sites and thereafter overnight at 4°C with the primary antibody in PBS containing 0.3% Tween-20 and 5% GS used at the following dilutions: anti-PAR (1/250), anti-OX-42 (1/150), anti-ED-1 (1/150), anti-BNDF (1/100). For BDNF detection, pre-treatment with proteinase K was used for antigen retrieval (10 µg/ml, 10 min). For cellular localization of PAR and BDNF expression in neurons and microglia, double immunohistofluorescence experiments have been performed by the simultaneous incubation of either the rabbit polyclonal antibodies recognizing PAR or BDNF and the mouse monoclonal antibodies recognizing NeuN or OX-42 at 4°C overnight. After 4 washes, antibody visualization was achieved by the incubation for 3 h at room temperature with Alexa 488-conjugated anti-mouse and/or Alexa 568-conjugated anti-rabbit IgGs (1∶1000). Negative controls were prepared by omitting the primary antibodies. The sections were then coverslipped with a fluorescent mounting medium and observed with an epifluorescent microscope (Eclipse E600, Nikon).

### Treatment

The 3-aminobenzamide (3-AB) was dissolved in DMSO (1 mg/µl). The DMSO solution was then diluted (1/100) in NaCl 0.9%. The 3-AB (90 mg/kg) was administered by i.p. route just after the onset of ischemia. The effects of 3-AB treatment were measured at different time points between 4 h and 1 month post-ictus and compared with those of corresponding vehicle.

### Quantification and Statistical Analysis

All assessments were performed by an observer blinded to the experimental conditions. The OX-42 immunoreactive cells were counted in the infarcted cortical area at 20× magnification using an epifluorescence microscope in 3 coronal sections at −1.60, −1.00, −0.40 mm from the bregma. Cells were counted continuously in 8 horizontal and 6 vertical fields passing by the center of the lesion using a 0.090 mm^2^ square grid. The final results were expressed per mm^2^.

All values were expressed as means±S.E.M. of n rats (six to eleven animals in each group). Comparisons among groups of rats were made using non-parametric Kruskal–Wallis test followed by Mann–Whitney for independent variables. Statistical significance was set at P<0.05.

## Results

### Infarct Volume and PARP-1 Activation

The infarct volume measurements were performed 4 h, 24 h, 8 d, 15 d and 30 d after the onset of ischemia. As shown in figure 2, photothrombotic ischemia leads in the early times (4 and 24 h) to an enlargement of the cortical lesion reaching a maximal value of 24,25 mm^3^ at 24 h. Consistent with previous data [40], [42], this first phase corresponds to the maturation of the infarction whereas at longer time points (8, 15 and 30 d), the infarct volumes decrease time-dependently until 3,90 mm^3^ at 30 d, the last time point considered in our study.

**Figure 3:**
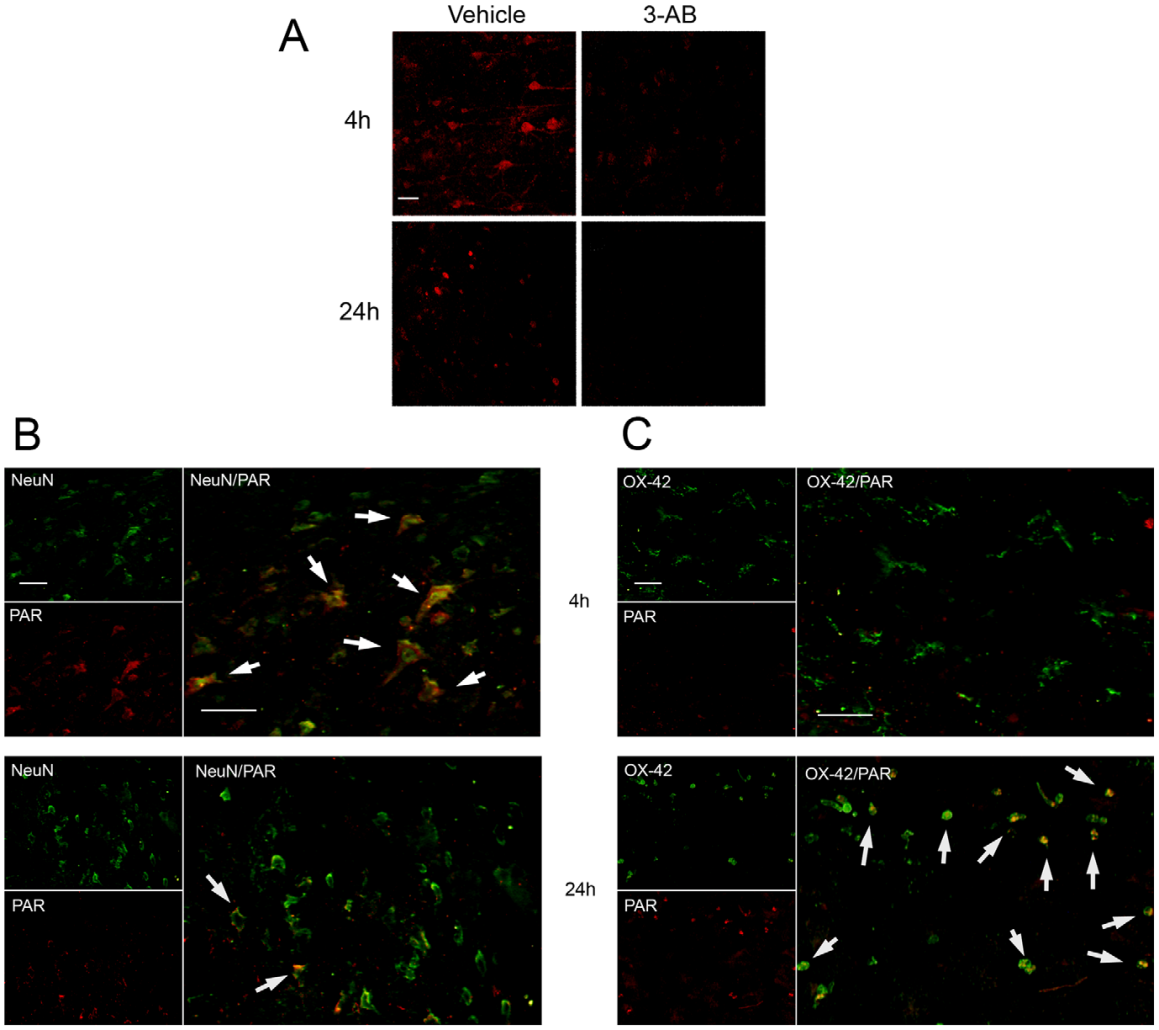
Effect of 3-AB treatment on PARP-1 activation and PAR/NeuN, PAR/OX-42 immunocolocalization. (A) PARP-1 activation, evidenced by PAR immunostaining, was performed on coronal sections of rats submitted to 4 h and 24 h of ischemia and treated with vehicle or with 3-AB. A marked decrease in PAR formation was observable after 3-AB treatment. (B) Immunocolocalization for PAR and NeuN was performed at 4 h and 24 h of ischemia. Arrows show double-positive cells. Ischemia-induced PAR formation in neurons is predominant at 4 h. (C) Immunocolocalization for PAR and OX-42 was performed at 4 h and 24 h of ischemia. Arrows show double-positive cells. Ischemia induces PAR formation in OX-42 positive cells at 24 h only. Photomicrographs are representative of 6 to 11 animals at each time point. Scale bars = 50 µm.

In our experimental conditions, photothrombotic ischemia leads to PARP-1 activation at 4 h and 24 h after the onset of ischemia as evidenced by a marked PAR immunostaining, the enzymatic product of PARP-1 (Fig. 3A). The staining was found maximal at 4 h as compared to 24 h of ischemia and disappeared at longer time points (data not shown). Cellular localization of PAR formation was assessed in neurons and microglia by double immunostaining experiments (Fig. 3B and 3C, respectively). Colocalization for PAR and NeuN revealed that PAR formation in neurons was found maximal at 4 h of ischemia as compared to 24 h (Fig. 3B). Conversely, PAR formation in microglial cells appeared only after 24 h of ischemia (Fig. 3C). Indeed, no PAR/OX-42 double-labeled cells were observable at 4 h of ischemia.

Consistent with a rapid PARP-1 activation described after focal ischemia [43], the PARP-1 inhibition was performed by 3-AB i.p. injection right after the induction of photothrombotic ischemia. Treatment with 3-AB produced an important decrease in PAR immunostaining at 4 h and resulted in an almost disappearance of poly(ADP-ribose) at 24 h (Fig. 3A), which demonstrates a reduced capacity for PARP-1 activation in 3-AB treated animals and validates our pharmacological treatment. Despite this effective inhibition, the 3-AB treatment (90 mg/kg, i.p.) did not produce any significant reduction in infarct volume and thus, whatever the kinetic points considered. Finally, to ascertain the absence of neuroprotection in 3-AB treated animals, the neuronal density was also assessed at 24 h. There was no variation between the two groups of animals (data not shown).

### OX-42 and ED-1 Immunostaining

#### OX-42

**Figure 4:**
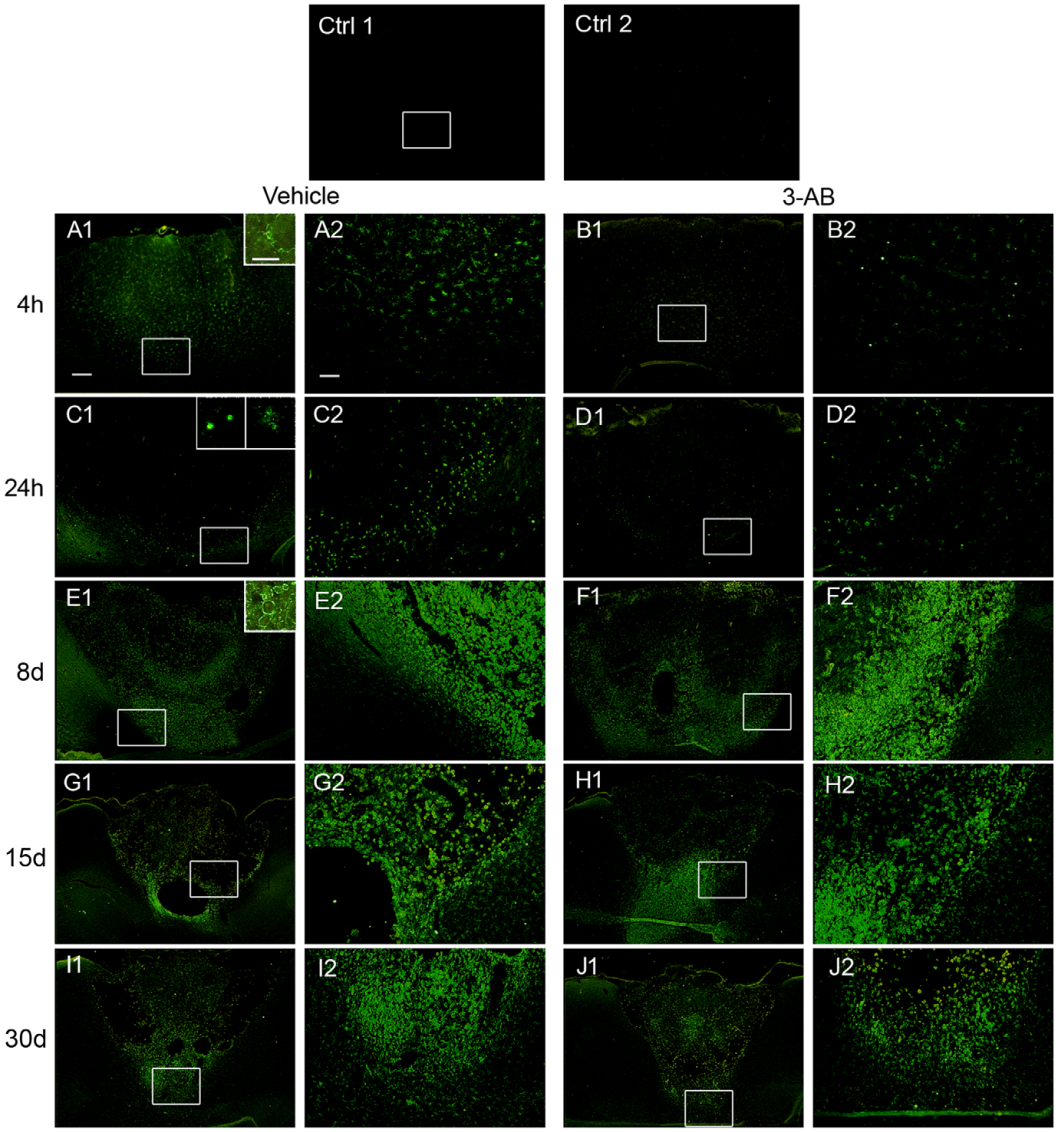
Time course of microglial/macrophages activation after ischemia: effect of 3-AB treatment. Microglial/macrophages activation was assessed at 4 h, 24 h, 8 d, 15 d and 30 d of photothrombotic ischemia by immunostaining using anti-OX-42 antibody, recognizing type 3 complement receptors. Rats treated with 3-AB showed an important decrease in microglial/macrophages activation in the acute phase of ischemia (B1–2 and D1–2). At longer time points, no differences were observed between vehicle (E, G, I) and 3-AB treated animals (F, H, J). Photomicrographs are representative of 6 to 11 animals at each time point. Scale bar = 200 µm in panels 1, 40 µm in panels 2 and 50 µm (insert).

Microglia/macrophages activation was assessed by OX-42 expression. As shown in figure 4, our data revealed that ischemia induces a robust microglia/macrophages activation observable as soon as 4 h, the earliest time point investigated in our study. At this time, OX-42 staining evidenced positive cells with a ramified morphology, homogenously distributed in the lesion (Fig. 4A1–2). After 24 h of ischemia, the staining was located in the boundary of the lesion and indicated the presence of two distinct cellular morphologies, stellate-shaped and rounded amoeboid cells (Fig. 4C1–2). At longer time points (8, 15 and 30 d), OX-42-positive cells exposed an amoeboid cellular morphology only and populated the whole infarcted area (Fig. 4E, G, I). Since microglia and hematogenous macrophages can adopt both ramified and amoeboid appearance [6], these two cellular types are undistinguishable as far as no discriminating cellular markers are available. However, based on studies using toxic liposomes depleting hematogenous macrophages [5] or GFP transgenic bone marrow chimeric mice [6], [7] we can assume that in our experimental conditions, early OX-42 and ED-1 staining observed in a very hypo-perfused area is attributable to local resident microglia while at longer time points (8, 15 and 30 d), these markers can be expressed by both types of cells.

In our experimental conditions, PARP-1 inhibition by 3-AB treatment was associated with a very important decrease in OX-42 staining at 4 h (Fig. 4B1–2) and 24 h (Fig. 4D1–2) of ischemia as compared to vehicle treated animals. Indeed, the number of OX-42 positive cells was significantly reduced by ∼75% at 4 h and ∼63% at 24 h of ischemia following 3-AB treatment (Table 1). These results showed that PARP-1 inhibition induces a robust decrease in resident microglial cells activation. On the other hand, no differences were observable after 8 d of ischemia between vehicle and 3-AB treated groups of animals (Fig. 4E, F, G, H, I, J and table 1).

**Table 1:** Number of OX-42 positive cells.

Time of ischemia	4 h	24 h	8 d	15 d	30 d
Vehicle	208±20	113±16	945±32	460±53	320±36
3-AB	54±12*	42±8*	938±45	539±39	262±42

Number of OX-42 positive cells after 4 h, 24 h, 8 d, 15 d and 30 d of ischemia in the infarcted area. Data represented the number of OX-42 positive cells par mm^2^ and are expressed as means±S.E.M. from 6 to 11 animals.

* P<0.05 vs vehicle treated animals.

#### ED-1

**Figure 5:**
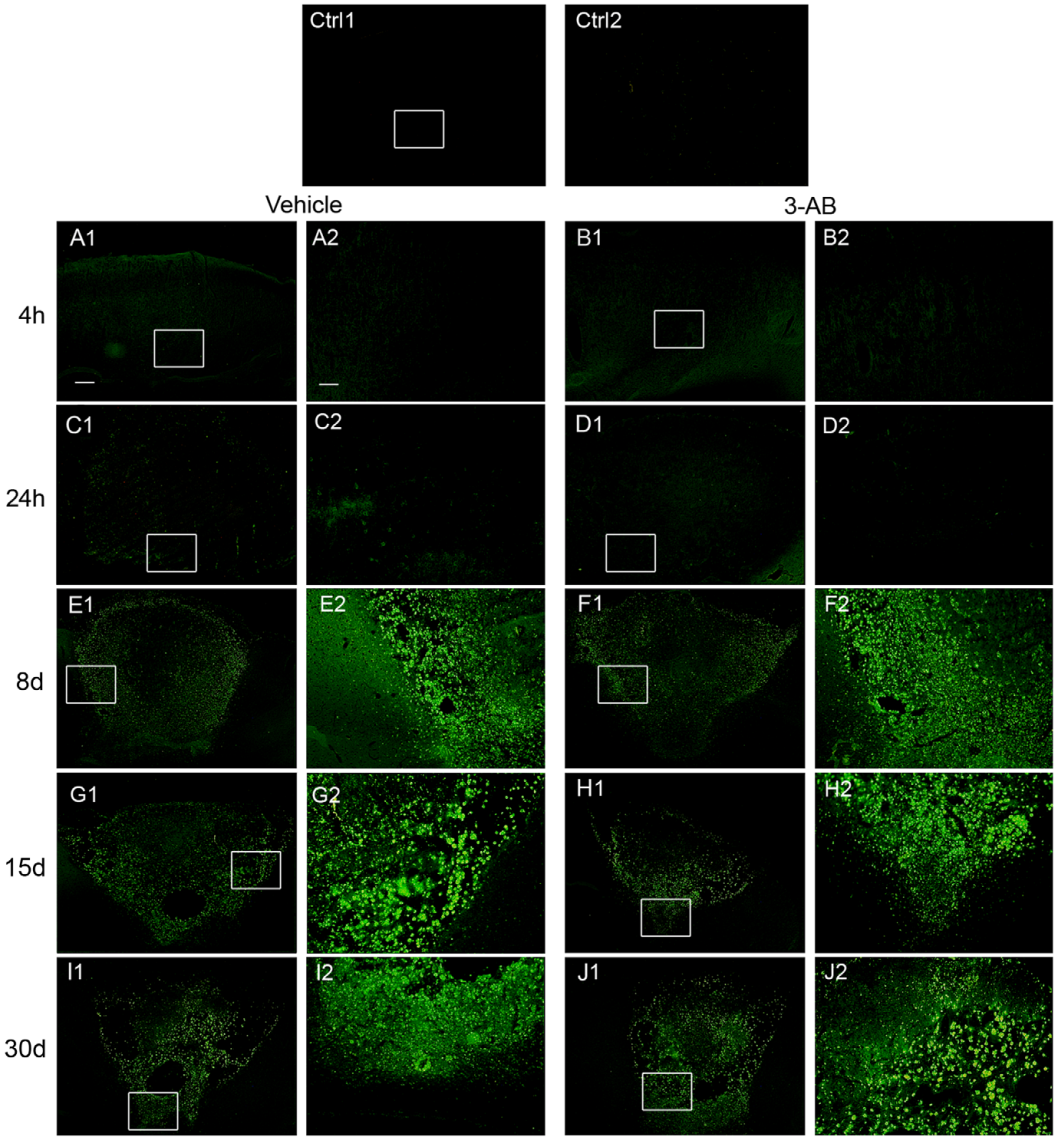
Time course of microglial/macrophages phagocytic activity after ischemia: effect of 3-AB treatment. Immunostaining for phagocytic activity (microglia/macrophages) was performed using anti-ED1 antibody at 4 h, 24 h, 8 d, 15 d and 30 d of photothrombotic ischemia. Note the absence of staining at 4 h in vehicle (A1–2) and 3-AB treated animals (B1-2). Rats treated with 3-AB (D1–2) showed a decrease in ED-1 staining at 24 h as compared to vehicle treated animals (C1–2). At longer time points, no differences were observed between vehicle (E, G, I) and 3-AB treated animals (F, H, J). Photomicrographs are representative of 6 to 11 animals at each time point. Scale bar = 200 µm in panels 1 and 40 µm in panels 2.

Because round amoeboid morphology can be associated with phagocytic capacity of activated local microglia and blood-born monocytes derived macrophages, the expression of lysosomal protein ED-1 was studied (Fig. 5). Our data show almost no ED-1 immunostaining in the early times of ischemia (4 h, 24 h; Fig. 5A1–2, C1–2), which is in agreement with the lack of phagocytic activity known during the ramified state in OX-42 positive cells. In the line with the few amoeboid cells observed after OX-42 staining in the vehicle treated group at 24 h, a few number of ED-1 positive cells were detected at this time of ischemia (Fig. 5C1–2). At longer time points, the pattern of ED-1 staining was similar to that observed with OX-42 marker with an intense staining within the infarcted zone. The similar distribution between these two markers reveals that most of the amoeboid cells have acquired phagocytic properties.

Treatment with 3-AB did not produce important differences in term of ED-1 expression. In the early times, we were able to detect the disappearance of the few ED-1 cells stained at 24 h (Fig. 5D1–2) whereas at longer time points, no marked differences were observable between vehicle and 3-AB treated groups of animals (Fig. 5E, F, G, H, I, J).

### GAP-43 and Synaptophysin Expression

Since neuroplastic changes have been shown to be a delayed phenomenon [44], GAP-43 and synaptophysin expression were investigated by western blotting experiments in the two concentric samples of cortical tissue P1 and P2 at longer time points of ischemia (8, 15 and 30 d).

**Figure 6:**
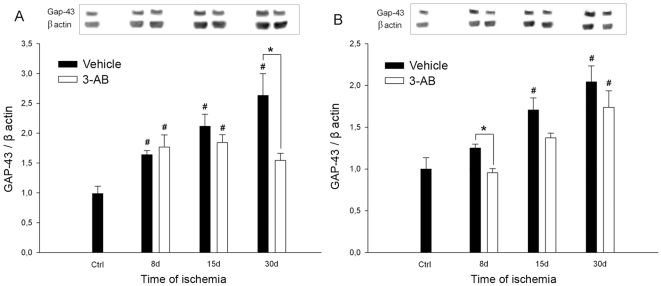
Time course of GAP-43 expression after ischemia: effect of 3-AB treatment. Western blots were prepared after 8 d, 15 d and 30 d of ischemia from P1 (A) and P2 (B) samples. Representative immunoblots are shown and densitometric analysis was performed after normalization of GAP-43 expression on β-actin levels. Photothrombotic ischemia induces an increase in GAP-43 expression from 8 d of ischemia in P1 and 15 d in P2. 3-AB treatment repressed GAP-43 expression in P1 at 30 d. In P2, the overall GAP-43 expression was lower in 3-AB treated animals but a significant decrease was found at 8 d only. Values are expressed as means±S.E.M. and are representative of 5 to 6 animals. (^#^ P<0.05 vs ctrl (sham operated animals); *P<0.05 vs vehicle treated animals).

As shown in figure 6, photothrombotic ischemia led to a significant and sustained increase in post-ischemic GAP-43 expression at 8, 15 and 30 d in P1 (Fig. 6A) and at 15 and 30 d in P2 (Fig. 6B) as compared to control values.

After 3-AB treatment, our results indicated that the expression of GAP-43 was significantly reduced in both P1 and P2 punches as compared to vehicle treated group of animals. The expression of GAP-43 was found significantly repressed in P1 at 30 d (−41%, Fig. 6A) and in P2 at 8 d only (−23%, Fig. 6B).

**Figure 7:**
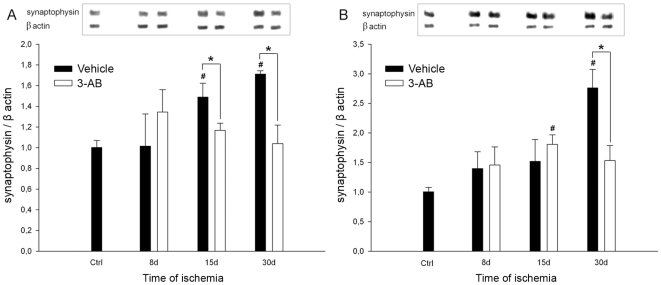
Time course of synaptophysin expression after ischemia: effect of 3-AB treatment. Western blots were prepared after 8 d, 15 d and 30 d of ischemia from P1 (A) and P2 (B) samples. Representative immunoblots are shown and densitometric analysis was performed after normalization of synaptophysin expression on β-actin levels. Photothrombotic ischemia induces an increase in synaptophysin expression in P1 after 15 d and 30 d of ischemia and in P2 after 30 d. 3-AB treatment abrogates synaptophysin induction in P1 after 15 d and 30 d of ischemia and in P2 after 30 d. Values are expressed as means±S.E.M. and are representative of 5 to 6 animals. (^#^ P<0.05 vs ctrl (sham operated animals); *P<0.05 vs vehicle treated animals).

Concerning synaptophysin expression, our results also showed that photothrombosis led to a significant increase in this protein expression in P1 at 15 and 30 d (Fig. 7A) and in P2 at 30 d (Fig. 7B) as compared to control values.

In 3-AB treated animals, the increase in synaptophysin expression was abrogated in both P1 and P2 cortical samples. The expression was found significantly lower in P1 at 15 and 30 d (−21% and −39%, respectively, Fig. 7A) and in P2 at 30 d (−44%, Fig. 7B) as compared to vehicle treated animals.

### BDNF Expression

#### ELISA

**Figure 8:**
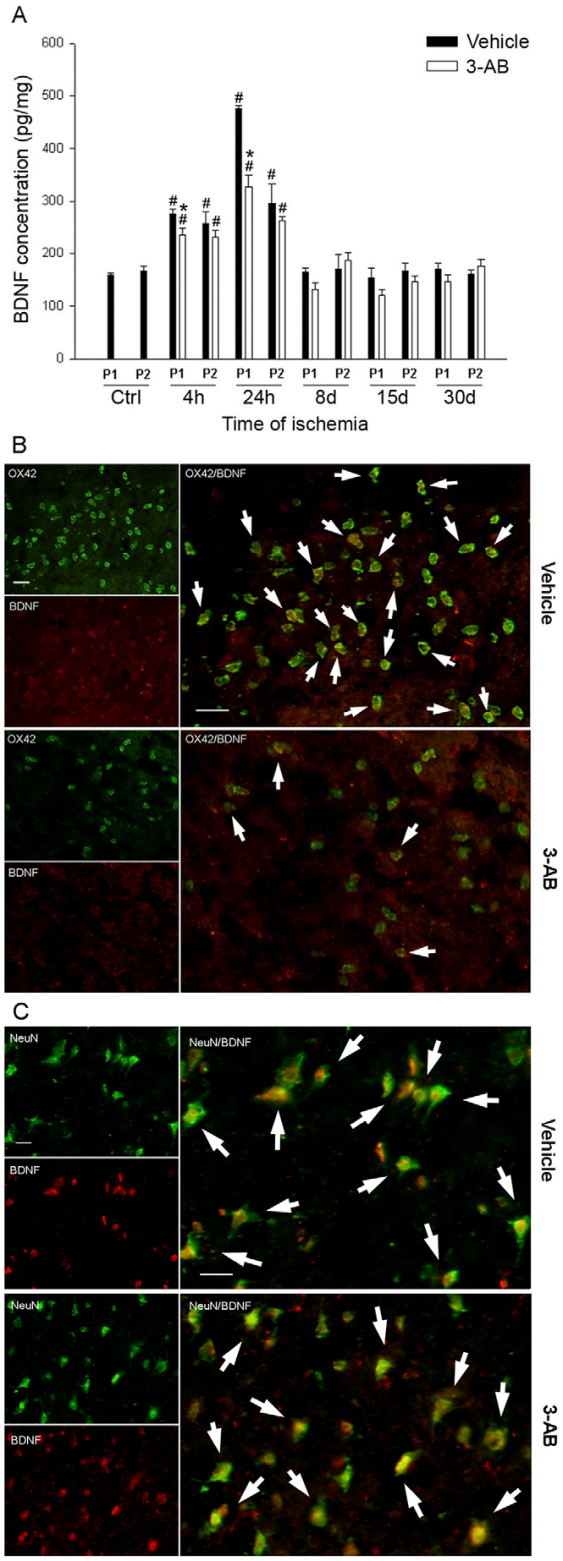
Time course of BDNF production after ischemia and BDNF/OX-42 immunocolocalization: effect of 3-AB treatment. (A) Elisa measurements of BDNF production in P1 and P2 samples were performed at 4 h, 24 h, 8 d, 15 d and 30 d of ischemia. Photothrombosis induces a significant increase in BDNF production in both P1 and P2 at 4 h and 24 h of ischemia. Note that the production was found maximal in P1 at 24 h. Rats treated with 3-AB showed a significant reduction in BDNF production in P1 at 4 h and 24 h of ischemia. Values are expressed as means±S.E.M. and are representative of 5 to 6 animals. (^#^ P<0.05 vs ctrl (sham operated animals); *P<0.05 vs vehicle treated animals). (B) Immunocolocalization for OX-42 and BNDF was performed at 24 h of ischemia. Arrows show double-positive cells. Ischemia induces expression of BDNF in some OX-42 - positive cells. Rats treated with 3-AB showed an almost complete disappearance of double positive cells. (C) Immunocolocalization for NeuN and BDNF was performed at 24 h of ischemia. Arrows show double-positive cells. Ischemia induces expression of BDNF in neurons. Rats treated with 3-AB exhibited a similar co-immunostaining. Photomicrographs are representative of 6 to 11 animals. Scale bars = 50 µm.

The ELISA assessment of tissue BDNF level was performed in each cortical punch (P1/P2, Fig. 8A). Our data revealed that photothrombotic ischemia induces an early increase in BDNF production in these two samples. As compared to control levels (160 and 167 pg/mg in P1 and P2, respectively), post-ischemic BDNF production increased significantly by 71% and 53% at 4 h and by 296% and 176% at 24 h. After 8 days of ischemia, the cortical level of BDNF returned to control values.

Following 3-AB treatment, the increase in BDNF levels was significantly attenuated in the lesioned area P1 only, after both 4 and 24 h of ischemia. The reduction was modest at 4 h (12%) whereas it reached 32% after 24 h of ischemia as compared to vehicle treated animals.

#### BDNF and OX-42 immunocolocalization

To further evaluate the role of microglia in BDNF production, immunohistofluorescence for OX-42 and BDNF expression was performed after 24 h of ischemia. As shown in figure 8B, colocalization experiments indicated that OX-42-positive cells located in the lesioned tissue, expressed BDNF. Nevertheless, the double immunostaining revealed that the colocalization was only partial since only a third of OX-42-positive cells produced BDNF.

As already mentionned, 3-AB treatment greatly reduced OX-42 immunostaining. Accordingly, BDNF-positive microglial cells were hardly identified as compared to vehicle treated animals.

#### BDNF and NeuN immunocolocalization

Neuronal expression of BDNF was assessed by immunohistofluorescence for NeuN and BDNF after 24 h of ischemia. As shown in figure 8C, colocalization experiments indicated that most of the remaining neurons in the lesioned tissue expressed BDNF. Following 3-AB treatment, a similar pattern of expression was found as compared to vehicle treated animals (Fig. 8C).

## Discussion

Our study reveals that the decrease in acute microglial activation was associated with a long term down regulation of synaptophysin and GAP-43 expression and to a significant decrease in BDNF production. Taken together, our data argue in favour of an important role for microglia in brain neuroplasticity stimulation possibly through BDNF production.

In order to investigate the role of microglial cells in the stimulation of neuroplastic changes, PARP-1 inhibition was used as a means of down-regulating the microglial response induced by ischemia. PARP-1 is a nuclear enzyme that normally functions to facilitate DNA repair. It is also well established that extensive PARP-1 activation promotes cell death through processes involving energy depletion and the release of apoptosis-inducing factor [45]–[47]. In our study, the choice of a strategy targeting PARP-1 activation was based on previous studies showing that PARP-1 is also an essential component in the regulation of inflammatory response through its function as a coactivator of NF-κB [37], [48], [49]. Our data show that photothrombotic focal ischemia was associated with an early PARP-1 activation occurring within the first 24 h with a maximal intensity observed at 4 h after the onset of ischemia which is consistent with previous reports [50]. Double immunostaining for PAR and NeuN or OX-42 revealed that PAR formation in neurons was an early process as compared to that observed in microglial cells. Indeed, PAR formation in neurons was visible with a maximal intensity after 4 h whereas it appeared only after 24 h of ischemia in microglial cells. Our results are in agreement with previous data showing a rapid PARP-1 activation in neurons after a focal brain ischemia [43] and are also consistent with previous reports showing an activation of PARP-1 in microglial cells [19], [37]. Interestingly, despite the effective PARP-1 inhibition evidenced by a strong decrease in PAR staining, no significant differences in term of temporal evolution of lesion size between vehicle and 3-AB treated animals were observed. Noteworthy, the relative high dose of 3-AB (90 mg/kg, i.p.) was purposely used in our experimental conditions in order to target the inflammatory response only. Such a choice was initially based on study showing that 3-AB is no more neuroprotective beyond 80 mg/kg (i.p.) following MCAO in mice [51]. Indeed, since the inflammatory reaction intensity is closely related to the extent of the lesion, the investigation of the role of microglial cells activation would have been inappropriate if a marked decrease in the inflammatory reaction was found to be associated with a strong and significant neuroprotection. The lack of protective effects observed in studies using high doses of 3-AB and its derivatives is not fully understood. It has been suggested that benzamide could produce non-specific effects counteracting with glucose metabolism, nucleic acid and protein synthesis [36]. However, since similar U-shaped dose-response curve has been observed with structurally distinct PARP inhibitors such as 3,4-dihydro 5-[4-(1-piperidinyl) butoxy]-1(2*H*)-isoquinolinone (DPQ) in response to cerebral ischemia [52], it can be hypothesized that high doses of PARP-1 inhibitors could impair DNA repair in the peri-infarcted area that otherwise would have been rescued. Finally, a robust and important decrease of OX-42 and ED-1 staining was observed in 3-AB treated animals in the early phase of ischemia, confirming that PARP-1 inhibition is an efficient approach for the modulation of local resident microglial activation and proliferation.

One of the most striking finding of this work is that the repression of microglial activation and proliferation in the acute stage of ischemia is associated with a significant decrease in the long term expression of neuronal plasticity proteins in the vicinity of the lesioned area. Indeed, the expression of GAP-43, an intracellular growth protein playing a role in sprouting, neuronal pathfinding and branching [53] and the expression of synaptophysin, an integral membrane protein of synaptic vesicles, widely used as an immunohistological marker for the determination of synaptogenesis [54] were both significantly reduced in 3-AB treated animals as compared to vehicle treated animals. Concerning GAP-43 expression, 3-AB treatment induces a reduced expression already significant at 8 d and at one month post-ischemia whereas statistical differences were found for synaptophysin expression at longer time points (15 and 30 d of ischemia) between the two groups of animals. Thus, our results suggest that limiting the early microglial activation is associated with a decrease in neurite outgrowth and synaptogenesis that normally occurred after brain injuries [44], [55]–[57]. This raises the possibility that microglial cells play an important role in the reorganization of the tissue surrounding the lesioned area.

Among the neurosupportive coupounds synthesized by microglia, experimental evidences point out to BDNF as an appealing candidate. In the past decade, BDNF has been involved in learning, memory and neuroplasticity [58], [59]. In addition, exogenous treatment with BDNF or exposing animals to enriched housing or exercise regimens, which also increases BDNF, enhances behavioral recovery after brain injury [33], [60], [61]. Moreover, blocking BDNF through antisense or immunoadhesin chimera (TrkB-IgG) strategies negates exercice-induced cognitive improvement after focal ischemia [62] or traumatic brain injury [63]. As assessed by ELISA, our study showed that photothrombotic ischemia is associated with a significant and robust increase in BDNF production within the infarct zone at 4 h and 24 h of ischemia. This result is consistent with an early increase in BDNF levels in the hemisphere ipsilateral to a focal photothrombotic lesion in rats [64]. In addition, our immunohistological experiments specify for the first time after a focal ischemia, that approximately a third of microglial cells express BDNF after 24 h of photothrombotic ischemia. Concerning this partial BDNF expression, it can be hypothesized that among microglial cells populating the brain lesion, coexist several subpopulations with opposite phenotypes. This hypothesis is conceivable regarding the highly complex (ambivalent) microglial response in damage formation, inflammation and repair after stroke. Following 3-AB treatment, BDNF levels are significantly decreased by 30% and microglial cells expressing BDNF hardly identified as compared to vehicle treated animals. This only ∼30% decrease in BDNF levels as compared to the two-third decrease in microglial cells number in 3-AB treated animals can be explained by the fact that BDNF remains expressed by neurons. Consistently, our immunohistological experiments reveal that both groups of animals exhibit a similar BDNF staining in neurons. Such a neuronal expression of BDNF is in agreement with previous studies reporting that brain ischemia results in an early induction of this trophic factor as evidenced by upregulation of BDNF mRNA or protein levels in neurons within and around the lesion [64]–[66].

Although not investigated directly here, BDNF production may be coupled to GAP-43 and synaptophysin inductions in several ways. Indeed, in a model of cervical axotomy, BDNF injection has been reported to stimulate GAP-43 expression and consequently axogenesis and repair [67]. Moreover, a very recent study performed on cortical culture using GAP-43 knockout animals directly shows that GAP-43 is necessary to the neurotrophic effects of BDNF [68]. In this study, GAP-43 was identified as a common mediator of the plastic regeneration effect of BDNF. At the synaptic level, BDNF has been shown to be important for synaptogenesis and to synaptic ultra structural composition in developing and adult brain [35], [69]. In addition, treatment using statin that induces BDNF up-regulation is linked to an increase in synaptophysin expression and to a significant improvement in functional recovery [55]. Consistently, blocking BDNF action abrogates exercice-induced synaptophysin expression [70]. Likewise, BDNF knockout mice have a reduced level of synaptophysin in hippocampal synaptosomes [71]. Finally, even though our study and others suggest that BDNF is a preponderant compound in mediating post-ischemic neuronal plasticity processes, it is important to underline that other molecules besides BDNF such as IGF-1 [22], [24], GDNF [72], [73], thrombospondins [74] or erythropoietin [75], [76], which are also produced by microglial cells, have been reported to promote post-stroke plasticity events. Thus, it is likely that neuroplasticity processes are orchestrated by several compounds, BDNF being one of these pro-neuroplastic factors.

In conclusion, the present findings imply a supportive role for microglial cells in the induction of neuroplastic changes after ischemia through the production of BDNF. Although important progress has been made in the comprehension of microglial cells function, the identification of the mechanisms that determine microglia accomplishing destructive or constructive role in the CNS is a prerequisite in elaborating future strategies that would maintain and sustain the trophic support provided by microglial cells. A targeted protection of neurosupportive microglial cells could represent a novel and exciting approach to potentiate post-stroke neuroregenerative responses.

## References

[1] Dirnagl U, Iadecola C, Moskowitz MA (1999). Pathobiology of ischaemic stroke: an integrated view.. Trends Neurosci.

[2] Kempermann G, Neumann H (2003). Neuroscience. Microglia: the enemy within?. Science.

[3] Brea D, Sobrino T, Ramos-Cabrer P, Castillo J (2009). Inflammatory and neuroimmunomodulatory changes in acute cerebral ischemia.. Cerebrovasc Dis.

[4] Kreutzberg GW (1996). Microglia: a sensor for pathological events in the CNS.. Trends Neurosci.

[5] Schroeter M, Jander S, Huitinga I, Witte OW, Stoll G (1997). Phagocytic response in photochemically induced infarction of rat cerebral cortex. The role of resident microglia.. Stroke.

[6] Schilling M, Besselmann M, Leonhard C, Mueller M, Ringelstein EB (2003). Microglial activation precedes and predominates over macrophage infiltration in transient focal cerebral ischemia: a study in green fluorescent protein transgenic bone marrow chimeric mice.. Exp Neurol.

[7] Schilling M, Besselmann M, Muller M, Strecker JK, Ringelstein EB (2005). Predominant phagocytic activity of resident microglia over hematogenous macrophages following transient focal cerebral ischemia: an investigation using green fluorescent protein transgenic bone marrow chimeric mice.. Exp Neurol.

[8] Feuerstein GZ, Wang X (2001). Inflammation and stroke: benefits without harm?. Arch Neurol.

[9] Marchetti B, Abbracchio MP (2005). To be or not to be (inflamed)–is that the question in anti-inflammatory drug therapy of neurodegenerative disorders?. Trends Pharmacol Sci.

[10] Ekdahl CT, Kokaia Z, Lindvall O (2009). Brain inflammation and adult neurogenesis: the dual role of microglia.. Neuroscience.

[11] Hanisch UK (2002). Microglia as a source and target of cytokines.. Glia.

[12] Zheng Z, Yenari MA (2004). Post-ischemic inflammation: molecular mechanisms and therapeutic implications.. Neurol Res.

[13] Chong ZZ, Li F, Maiese K (2005). Oxidative stress in the brain: novel cellular targets that govern survival during neurodegenerative disease.. Prog Neurobiol.

[14] Tuttolomondo A (2008). Citokines and inflammation markers in ischemic stroke.. Curr Pharm Des.

[15] Block ML, Zecca L, Hong JS (2007). Microglia-mediated neurotoxicity: uncovering the molecular mechanisms.. Nat Rev Neurosci.

[16] Yrjanheikki J, Tikka T, Keinanen R, Goldsteins G, Chan PH (1999). A tetracycline derivative, minocycline, reduces inflammation and protects against focal cerebral ischemia with a wide therapeutic window.. Proc Natl Acad Sci U S A.

[17] Tikka T, Fiebich BL, Goldsteins G, Keinanen R, Koistinaho J (2001). Minocycline, a tetracycline derivative, is neuroprotective against excitotoxicity by inhibiting activation and proliferation of microglia.. J Neurosci.

[18] Ekdahl CT, Claasen JH, Bonde S, Kokaia Z, Lindvall O (2003). Inflammation is detrimental for neurogenesis in adult brain.. Proc Natl Acad Sci U S A.

[19] Kauppinen TM, Suh SW, Berman AE, Hamby AM, Swanson RA (2009). Inhibition of poly(ADP-ribose) polymerase suppresses inflammation and promotes recovery after ischemic injury.. J Cereb Blood Flow Metab.

[20] Neumann J, Gunzer M, Gutzeit HO, Ullrich O, Reymann KG (2006). Microglia provide neuroprotection after ischemia.. Faseb J.

[21] Imai F, Suzuki H, Oda J, Ninomiya T, Ono K (2007). Neuroprotective effect of exogenous microglia in global brain ischemia.. J Cereb Blood Flow Metab.

[22] Lalancette-Hebert M, Gowing G, Simard A, Weng YC, Kriz J (2007). Selective ablation of proliferating microglial cells exacerbates ischemic injury in the brain.. J Neurosci.

[23] Kim BJ, Kim MJ, Park JM, Lee SH, Kim YJ (2009). Reduced neurogenesis after suppressed inflammation by minocycline in transient cerebral ischemia in rat.. J Neurol Sci.

[24] Thored P, Heldmann U, Gomes-Leal W, Gisler R, Darsalia V (2009). Long-term accumulation of microglia with proneurogenic phenotype concomitant with persistent neurogenesis in adult subventricular zone after stroke.. Glia.

[25] Nakajima K, Yamamoto S, Kohsaka S, Kurihara T (2008). Neuronal stimulation leading to upregulation of glutamate transporter-1 (GLT-1) in rat microglia in vitro.. Neurosci Lett.

[26] Stoll G, Jander S (1999). The role of microglia and macrophages in the pathophysiology of the CNS.. Prog Neurobiol.

[27] Neumann J, Sauerzweig S, Ronicke R, Gunzer F, Dinkel K (2008). Microglia cells protect neurons by direct engulfment of invading neutrophil granulocytes: a new mechanism of CNS immune privilege.. J Neurosci.

[28] Batchelor PE, Liberatore GT, Wong JY, Porritt MJ, Frerichs F (1999). Activated macrophages and microglia induce dopaminergic sprouting in the injured striatum and express brain-derived neurotrophic factor and glial cell line-derived neurotrophic factor.. J Neurosci.

[29] Lai AY, Todd KG (2008). Differential regulation of trophic and proinflammatory microglial effectors is dependent on severity of neuronal injury.. Glia.

[30] Nagamoto-Combs K, McNeal DW, Morecraft RJ, Combs CK (2007). Prolonged microgliosis in the rhesus monkey central nervous system after traumatic brain injury.. J Neurotrauma.

[31] Schabitz WR, Schwab S, Spranger M, Hacke W (1997). Intraventricular brain-derived neurotrophic factor reduces infarct size after focal cerebral ischemia in rats.. J Cereb Blood Flow Metab.

[32] Wu D (2005). Neuroprotection in experimental stroke with targeted neurotrophins.. NeuroRx.

[33] Schabitz WR, Steigleder T, Cooper-Kuhn CM, Schwab S, Sommer C (2007). Intravenous brain-derived neurotrophic factor enhances poststroke sensorimotor recovery and stimulates neurogenesis.. Stroke.

[34] Bessis A, Bechade C, Bernard D, Roumier A (2007). Microglial control of neuronal death and synaptic properties.. Glia.

[35] Mattson MP (2008). Glutamate and neurotrophic factors in neuronal plasticity and disease.. Ann N Y Acad Sci.

[36] Szabo C, Dawson VL (1998). Role of poly(ADP-ribose) synthetase in inflammation and ischaemia-reperfusion.. Trends Pharmacol Sci.

[37] Chiarugi A, Moskowitz MA (2003). Poly(ADP-ribose) polymerase-1 activity promotes NF-kappaB-driven transcription and microglial activation: implication for neurodegenerative disorders.. J Neurochem.

[38] Kauppinen TM, Swanson RA (2007). The role of poly(ADP-ribose) polymerase-1 in CNS disease.. Neuroscience.

[39] Van Hoecke M, Prigent-Tessier AS, Garnier PE, Bertrand NM, Filomenko R (2007). Evidence of HIF-1 functional binding activity to caspase-3 promoter after photothrombotic cerebral ischemia.. Mol Cell Neurosci.

[40] Van Hoecke M, Prigent-Tessier A, Bertrand N, Prevotat L, Marie C (2005). Apoptotic cell death progression after photothrombotic focal cerebral ischaemia: effects of the lipophilic iron chelator 2,2′-dipyridyl.. Eur J Neurosci.

[41] Laemmli UK (1970). Cleavage of structural proteins during the assembly of the head of bacteriophage T4.. Nature.

[42] Demougeot C, Van Hoecke M, Bertrand N, Prigent-Tessier A, Mossiat C (2004). Cytoprotective efficacy and mechanisms of the liposoluble iron chelator 2,2′-dipyridyl in the rat photothrombotic ischemic stroke model.. J Pharmacol Exp Ther.

[43] Tokime T, Nozaki K, Sugino T, Kikuchi H, Hashimoto N (1998). Enhanced poly(ADP-ribosyl)ation after focal ischemia in rat brain.. J Cereb Blood Flow Metab.

[44] Stroemer RP, Kent TA, Hulsebosch CE (1995). Neocortical neural sprouting, synaptogenesis, and behavioral recovery after neocortical infarction in rats.. Stroke.

[45] Ha HC, Snyder SH (1999). Poly(ADP-ribose) polymerase is a mediator of necrotic cell death by ATP depletion.. Proc Natl Acad Sci U S A.

[46] Ying W, Chen Y, Alano CC, Swanson RA (2002). Tricarboxylic acid cycle substrates prevent PARP-mediated death of neurons and astrocytes.. J Cereb Blood Flow Metab.

[47] Yu SW, Wang H, Poitras MF, Coombs C, Bowers WJ (2002). Mediation of poly(ADP-ribose) polymerase-1-dependent cell death by apoptosis-inducing factor.. Science.

[48] Oliver FJ, Menissier-de Murcia J, Nacci C, Decker P, Andriantsitohaina R (1999). Resistance to endotoxic shock as a consequence of defective NF-kappaB activation in poly (ADP-ribose) polymerase-1 deficient mice.. Embo J.

[49] Moroni F (2008). Poly(ADP-ribose)polymerase 1 (PARP-1) and postischemic brain damage.. Curr Opin Pharmacol.

[50] Takahashi K, Greenberg JH (1999). The effect of reperfusion on neuroprotection using an inhibitor of poly(ADP-ribose) polymerase.. Neuroreport.

[51] Couturier JY, Ding-Zhou L, Croci N, Plotkine M, Margaill I (2003). 3-Aminobenzamide reduces brain infarction and neutrophil infiltration after transient focal cerebral ischemia in mice.. Exp Neurol.

[52] Takahashi K, Pieper AA, Croul SE, Zhang J, Snyder SH (1999). Post-treatment with an inhibitor of poly(ADP-ribose) polymerase attenuates cerebral damage in focal ischemia.. Brain Res.

[53] Benowitz LI, Routtenberg A (1997). GAP-43: an intrinsic determinant of neuronal development and plasticity.. Trends Neurosci.

[54] Valtorta F, Pennuto M, Bonanomi D, Benfenati F (2004). Synaptophysin: leading actor or walk-on role in synaptic vesicle exocytosis?. Bioessays.

[55] Chen J, Zhang C, Jiang H, Li Y, Zhang L (2005). Atorvastatin induction of VEGF and BDNF promotes brain plasticity after stroke in mice.. J Cereb Blood Flow Metab.

[56] Carmichael ST (2006). Cellular and molecular mechanisms of neural repair after stroke: making waves.. Ann Neurol.

[57] Millerot-Serrurot E, Chausset A, Mossiat C, Prigent-Tessier A, Bertrand N (2007). Effect of early decrease in the lesion size on late brain tissue loss, synaptophysin expression and functionality after a focal brain lesion in rats.. Neurochem Int.

[58] Binder DK, Scharfman HE (2004). Brain-derived neurotrophic factor.. Growth Factors.

[59] Lipsky RH, Marini AM (2007). Brain-derived neurotrophic factor in neuronal survival and behavior-related plasticity.. Ann N Y Acad Sci.

[60] Vaynman S, Ying Z, Gomez-Pinilla F (2003). Interplay between brain-derived neurotrophic factor and signal transduction modulators in the regulation of the effects of exercise on synaptic-plasticity.. Neuroscience.

[61] Kim MW, Bang MS, Han TR, Ko YJ, Yoon BW (2005). Exercise increased BDNF and trkB in the contralateral hemisphere of the ischemic rat brain.. Brain Res.

[62] Ploughman M, Windle V, MacLellan CL, White N, Dore JJ (2009). Brain-derived neurotrophic factor contributes to recovery of skilled reaching after focal ischemia in rats.. Stroke.

[63] Griesbach GS, Hovda DA, Gomez-Pinilla F (2009). Exercise-induced improvement in cognitive performance after traumatic brain-injury in rats is dependent on BDNF Activation.. Brain Res.

[64] Sulejczak D, Ziemlinska E, Czarkowska-Bauch J, Nosecka E, Strzalkowski R (2007). Focal photothrombotic lesion of the rat motor cortex increases BDNF levels in motor-sensory cortical areas not accompanied by recovery of forelimb motor skills.. J Neurotrauma.

[65] Kokaia Z, Zhao Q, Kokaia M, Elmer E, Metsis M (1995). Regulation of brain-derived neurotrophic factor gene expression after transient middle cerebral artery occlusion with and without brain damage.. Exp Neurol.

[66] Rickhag M, Teilum M, Wieloch T (2007). Rapid and long-term induction of effector immediate early genes (BDNF, Neuritin and Arc) in peri-infarct cortex and dentate gyrus after ischemic injury in rat brain.. Brain Res.

[67] Kobayashi NR, Fan DP, Giehl KM, Bedard AM, Wiegand SJ (1997). BDNF and NT-4/5 prevent atrophy of rat rubrospinal neurons after cervical axotomy, stimulate GAP-43 and Talpha1-tubulin mRNA expression, and promote axonal regeneration.. J Neurosci.

[68] Gupta SK, Mishra R, Kusum S, Spedding M, Meiri KF (2009). GAP-43 is essential for the neurotrophic effects of BDNF and positive AMPA receptor modulator S18986.. Cell Death Differ.

[69] Waterhouse EG, Xu B (2009). New insights into the Role of Brain-derived Neurotrophic Factor in Synaptic Plasticity.. Mol Cell Neurosci.

[70] Vaynman SS, Ying Z, Yin D, Gomez-Pinilla F (2006). Exercise differentially regulates synaptic proteins associated to the function of BDNF.. Brain Res.

[71] Pozzo-Miller LD, Gottschalk W, Zhang L, McDermott K, Du J (1999). Impairments in high-frequency transmission, synaptic vesicle docking, and synaptic protein distribution in the hippocampus of BDNF knockout mice.. J Neurosci.

[72] Kobayashi T, Ahlenius H, Thored P, Kobayashi R, Kokaia Z (2006). Intracerebral infusion of glial cell line-derived neurotrophic factor promotes striatal neurogenesis after stroke in adult rats.. Stroke.

[73] Batchelor PE, Wills TE, Hewa AP, Porritt MJ, Howells DW (2008). Stimulation of axonal sprouting by trophic factors immobilized within the wound core.. Brain Res.

[74] Chamak B, Dobbertin A, Mallat M (1995). Immunohistochemical detection of thrombospondin in microglia in the developing rat brain.. Neuroscience.

[75] Bernaudin M, Marti HH, Roussel S, Divoux D, Nouvelot A (1999). A potential role for erythropoietin in focal permanent cerebral ischemia in mice.. J Cereb Blood Flow Metab.

[76] Wang L, Zhang Z, Wang Y, Zhang R, Chopp M (2004). Treatment of stroke with erythropoietin enhances neurogenesis and angiogenesis and improves neurological function in rats.. Stroke.

